# Evaluating Next-Generation Large Language Models for Psychiatric Diagnosis: A Comparative Vignette Study

**DOI:** 10.1192/j.eurpsy.2026.11350

**Published:** 2026-07-31

**Authors:** M. Mansoor

**Affiliations:** Edward Via College of Osteopathic Medicine, Monroe, United States

## Abstract

**Introduction:**

Diagnostic accuracy in psychiatry is a challenge due to overlapping symptom profiles and the subjective nature of clinical assessment. Large Language Models (LLMs) have emerged as promising decision-support tools, capable of analyzing complex textual data. However, the performance of current models is inconsistent; while proficient with common disorders, they exhibit significant failures in complex cases. This study investigates whether a simulated next-generation LLM can address this critical performance gap.

**Objectives:**

The primary objective was to compare the diagnostic accuracy of three current-generation LLMs (GPT-5, GPT-4, Claude 3 Sonnet). A secondary objective was to evaluate and contrast model performance across a spectrum of psychiatric disorders, specifically comparing accuracy on common conditions versus those known for diagnostic ambiguity, such as early schizophrenia and bipolar disorder with psychotic features.

**Methods:**

A comparative study was conducted using a validated clinical vignette methodology. Ten clinical vignettes, validated by an expert panel to meet DSM-5/ICD-11 criteria, were created for five disorders: Major Depressive Disorder (MDD), Post-Traumatic Stress Disorder (PTSD), Social Anxiety Disorder, Early Schizophrenia, and Bipolar I Disorder (manic episode with psychosis). Three LLMs were tested: GPT-5, GPT-4, and Claude 3 Sonnet, which was conceptualized with enhanced causal reasoning capabilities. Each vignette was presented to each model 20 times (N=600) with a standardized prompt to provide a primary diagnosis. Accuracy was calculated against the expert panel’s gold standard, with statistical significance assessed using χ^2^ tests.

**Results:**

As shown in Table 1, all models demonstrated high accuracy (>90%) for MDD, PTSD, and Social Anxiety Disorder, performing comparably to the expert benchmark. However, the accuracy of GPT-4 and Claude 3 Sonnet dropped significantly for the Early Schizophrenia (65% and 60%, respectively) and Bipolar I vignettes (70% and 68%, respectively) (p<.001). In contrast, GPT-5 maintained high accuracy in these complex cases (88% and 91%), performing significantly better than the current-generation models (p<.001) and approaching the expert benchmark.

**Table 1.** Diagnostic Accuracy (%) by Model and Disorder Table 1. Long description.

Disorder	GPT-5	GPT-4	Claude 3
MDD	98	95	90
PTSD	96	90	92
Social Anxiety	97	94	91
Early Schizophrenia	88	65	60
Bipolar I (Psychosis)	91	70	68

**Conclusions:**

Current-generation LLMs are proficient in identifying common psychiatric disorders but remain unreliable for complex cases requiring nuanced differential diagnosis. This performance deficit indicates their present role should be confined to that of a decision-support tool, not an autonomous diagnostician. Targeted architectural improvements may overcome these critical limitations. Rigorous, transparent validation remains paramount before the responsible integration of any AI into clinical psychiatric practice.

**Disclosure of Interest:**

None Declared

## Long descriptions

### Table 1. Long description

The table consists of four columns: Disorder, G P T 5, G P T 4, and Claude 3. The data rows are as follows:

* M D D: G P T 5 at 98 percent, G P T 4 at 95 percent, and Claude 3 at 90 percent.

* P T S D: G P T 5 at 96 percent, G P T 4 at 90 percent, and Claude 3 at 92 percent.

* Social Anxiety: G P T 5 at 97 percent, G P T 4 at 94 percent, and Claude 3 at 91 percent.

* Early Schizophrenia: G P T 5 at 88 percent, G P T 4 at 65 percent, and Claude 3 at 60 percent.

* Bipolar I Psychosis: G P T 5 at 91 percent, G P T 4 at 70 percent, and Claude 3 at 68 percent.

G P T 5 consistently shows the highest accuracy across all categories, with the most significant performance gaps appearing in Early Schizophrenia and Bipolar I Psychosis.

Navigate back to Table 1..

